# Mortality in Western Australian seniors with chronic respiratory diseases: a cohort study

**DOI:** 10.1186/1471-2458-10-385

**Published:** 2010-07-01

**Authors:** Kristjana Einarsdóttir, David B Preen, Frank M Sanfilippo, Raylene Reeve, Jon D Emery, C D'Arcy J Holman

**Affiliations:** 1 Centre for Health Services Research, School of Population Health, The University of Western Australia, 35 Stirling Highway, Crawley, 6009 Perth, Australia; 2 School of Primary, Aboriginal and Rural Health Care, The University of Western Australia, 35 Stirling Highway, Crawley, 6009 Perth, Australia

## Abstract

**Background:**

Relatively few studies have examined survival by pharmacotherapy level and the effects of patient characteristics on mortality by pharmacotherapy level in older chronic respiratory disease (CRD) patients. This study aimed to investigate these issues in older (≥ 65) CRD patients in Western Australia.

**Methods:**

We identified 108,312 patients ≥ 65 years with CRD during 1992-2006 using linked medical, pharmaceutical, hospital and mortality databases held by the Commonwealth and State governments. Pharmacotherapy classification levels were designed by a clinical consensus panel. Cox regression was used to investigate the study aim.

**Results:**

Patients using only short acting bronchodilators experienced similar, but slightly worse survival than patients in the highest pharmacotherapy level group using high dose inhaled corticosteroids (ICS) ± long acting bronchodilators (LABs) ± oral steroids. Patients using low to medium dose ICS ± LABs experienced relatively better survival. Also, male gender was associated with all-cause mortality in all patients (HR = 1.72, 95% CI 1.65-1.80) and especially in those in the highest pharmacotherapy level group (HR = 1.97, 95%CI = 1.84-2.10). The P-value of interaction between gender and pharmacotherapy level for the effect on all-cause death was significant (0.0003).

**Conclusions:**

Older patients with CRD not using ICS experienced the worst survival in this study and may benefit from an escalation in therapeutic regime. Males had a higher risk of death than females, which was more pronounced in the highest pharmacotherapy level group. Hence, primary health care should more actively direct disease management to mild-to-moderate disease patients.

## Background

Asthma and Chronic Obstructive Pulmonary Disease (COPD) account for 80% of the total burden of chronic respiratory diseases (CRD) in Australia [1] and represent a significant burden to the Australian health care system [2,3]. They are nevertheless under-diagnosed among the older population because of atypical presentation and co-morbidity [2,4,5]. These diseases have similar manifestations in older patients - wheezing, chest tightness and shortness of breath - despite very different causes [6,7]. The estimated prevalence of asthma in Australian adults over 15 years of age ranges from 10 to 12% [3], whereas approximately 9-12% of Australians over 45 years have symptomatic COPD [8,9].

The effects of patient demographic and clinical characteristics on mortality in the general population is well established [10]. For example, being a male, of indigenous race, of low socio-economic status (SES) or living in a remote area has been shown to increase the risk of dying from any cause [10]. However, relatively few studies have examined the risk factors for mortality in older patients (≥ 65) with asthma [11,12]. Also, the risk factors for mortality in older COPD patients have mainly been explored in patients who have been hospitalised for COPD [13,14]. This is perhaps surprising given relatively high prevalence of asthma and COPD, an ageing population demographic and substantial health care costs resulting from primary and secondary care of patients with these conditions [8,15,16]. Further, the effects of patient characteristics on mortality and how these effects differ according to pharmacotherapy level remains uncertain.

Treatment with inhaled corticosteroids (ICS) is the cornerstone of CRD management [17,18] despite that large randomly controlled trials have had difficulty in establishing a beneficial effect of ICS on survival in COPD patients [19]. Since distinguishing asthma and COPD in older patients with CRD is often challenging due to substantial overlap both in clinical manifestations and in the approach to disease management [6,7], studying the benefit of ICS on survival in older CRD patients overall remains important. We thus aimed to investigate (a) survival by pharmacotherapy level and (b) the effects of patient gender, race, SES, and residential remoteness on mortality due to any cause, overall as well as within groups of differing pharmacotherapy level, in older CRD patients in Western Australia (WA) during 1992-2006.

## Methods

### Data Sources

This study drew data from whole-population, de-identified hospital separation, mortality, pharmaceutical and medical claims databases linked and accessed through the Cross-Jurisdictional Linkage Facility of the WA Data Linkage System (WADLS) maintained by the WA Department of Health. The WADLS was established in 1995 and uses computerised probabilistic matching to link over 30 administrative health databases [20]. Matching procedures are based on full name and address, phonetic compression algorithms and other identifiers. An evaluation of the WADLS linkage has shown that the extensive matching procedures are 99.89% accurate [20].

We extracted linked data for WA residents aged at least 65 years between 1^st ^January 1992 and 31^st ^December 2006 from the Hospital Morbidity Data System (HMDS), the Death Registry, the Electoral Roll, the Medicare Benefits Scheme (MBS), and the Pharmaceutical Benefits Scheme (PBS). The HMDS data included principal and secondary diagnoses and procedures; and dates and types of admissions and separations. The Death Registry data included date and cause of death. The MBS data included dates and types of services provided by medical practitioners that qualified for Medicare benefits [21]. The PBS data included date of supply and type of medication prescribed. Registration information from the Electoral Roll was also extracted to ascertain inward and outward migration of the study population. Electoral Roll registration is compulsory for all Australian citizens residing in Australia who have reached 18 years of age.

### Study Population

Data records from the HMDS database, the death registry, MBS and PBS, for people who were at least 65 years of age at the time of record entry, were combined to ascertain the study population. We used the HMDS and death data to identify individuals with a diagnosis or death record of asthma, COPD or emphysema (ICD-9-CM: 492-493.92, 496, 975.7, E945.7; ICD-10-AM: J43-J46, T48.6, Y55.6) and bronchitis (ICD-9-CM: 491-491.21, 491.8-491.9; ICD-10-AM: J41-J42). The MBS data were used to identify individuals undergoing a special asthma management plan in general practitioner (GP) offices (asthma cycle of care) subsidised by the government (items 2546-2559 and 2664-2677). From the PBS data, a list of the most commonly used asthma/COPD medication in Australia was used to identify potential asthma/COPD patients. The drugs included beclomethasone, budesonide, fluticasone, eformoterol, salmeterol, terbutaline, salbutamol, ipratropium, tiotropium, nedocromil, theophylline and aminophylline. After combining these records, 116,983 individuals were identified having a HMDS, death, MBS and/or PBS asthma or COPD record. Individuals with acute bronchitis were excluded by removing all individuals with a bronchitis diagnosis or death record if they did not have any other record of an asthma or COPD-related diagnosis, treatment plan or medication (n = 386). We then excluded individuals who were not registered on the WA Electoral Roll during the study period (n = 8285). All other individuals were included in the study as asthma or COPD patients (n = 108,312). We refer to these conditions collectively as 'chronic respiratory diseases' (CRD) since they account for the majority of the CRD burden in Australia [1].

A clinical consensus panel comprising seven general practitioners, two geriatricians and three clinical pharmacists was convened to develop guidelines for classification of pharmacotherapy level (Table 1). If a patient had been dispensed a combination of medications from different levels, the patient would be assigned the level reflecting the highest pharmacotherapy ever given.

**Table 1:** Pharmacotherapy levels for CRD based on medication dose as defined by a clinical consensus panel

SABs	Low dose ICS *	Medium dose ICS ± LABs *	High dose ICS ± LABs ± OS *
Terbutaline AND/OR	< 250 μg Beclomethasone AND/OR	250-500 μg Beclomethasone AND/OR	> 500 μg Beclomethasone AND/OR
Salbutamol AND/OR	< 400 μg Budesonide AND/OR	400-800 μg Budesonide AND/OR	> 800 μg Budesonide AND/OR
Ipratropium AND/OR	< 250 μg Fluticasone	250-500 μg Fluticasone	> 500 μg Fluticasone
Tiotropium AND/OR			
Nedocromil		AND/OR	
		
		Eformoterol AND/OR	Eformoterol AND/OR
		Salmeterol	Salmeterol
			
			AND/OR
			
			> 1 prescription per year of Hydrocortisone and/or Prednisolone

SABs = Short Acting Bronchodilators

ICS = Inhaled Corticosteroids

LABs = Long Acting Bronchodilators

OS = Oral Steriods

* Doses represent daily doses

We used the Index of Relative Socio-Economic Disadvantage (IRD) to divide the study population into quintiles of SES disadvantage based on areas of residence. The variables used to compose the IRD are low income, low educational attainment and high unemployment and are collected as part of the Australian Census conducted every five years [22]. To describe geographical disadvantage, we used the Accessibility/Remoteness Index of Australia (ARIA +), which measures access in terms of physical distance from services and is grouped into five categories: major cities, inner regional, outer regional, remote, and very remote [23].

The study was approved by the Human Research Ethics Committees of The University of Western Australia and the WA Department of Health. It also conformed with the principles of the Declaration of Helsinki.

### Statistical Analyses

Patient follow-up began from the date of the first identified CRD record, start date of continuous Electoral Roll registration, or 1^st ^January 1992, whichever came last. Follow-up ended at the date of the last record of any of the HMDS/MBS/PBS databases, end date of the continuous Electoral Roll registration, date of death, or 31^st ^December 2006, whichever came first. We applied Cox proportional hazards models to calculate hazard ratios and 95% confidence intervals overall and by pharmacotherapy level. All models were adjusted for gender, age at start of follow-up, indigenous status, socio-economic status, residential remoteness, and Charlson index of co-morbidity [24]. We assessed the proportional hazards assumption for each covariate in each model and found no evidence against proportionality. We used Cox proportional hazards regression to estimate separate survival curves for each pharmacotherapy level with death due to any cause as outcome. The survival curves were adjusted for all variables mentioned above by using in the model the average value of each of the adjusting variables (mean of covariates method). All analyses were performed using SAS version 9.1 (SAS Institute Inc., Cary, NC, USA).

## Results

### Patient Characteristics

Of the 108,312 CRD patients in our study, 72% were selected on the basis of having had one or more CRD medication prescription, 3% were selected on the basis of a CRD hospital admission, 0.03% of having attended 'asthma cycle of care' GP services and 25% on the basis of having records from various combinations of the four datasets. Hence, the vast majority of our CRD patient cohort (97%) had been prescribed a CRD medication at least once.

Table 2 shows the characteristics of the 108,312 patients identified with CRD in our study, overall and by pharmacotherapy level. We observed 9,943 deaths during the 15 years of follow-up (average 6 years) with the majority being in the highest pharmacotherapy level group. The mortality rate was highest in the lowest (1,827.1 per 100,000 PY) and highest (1,826.3 per 100,000 PY) pharmacotherapy level groups. Mean age at start of follow-up in the whole group of patients was 72.7 years (SD 7.0), and was fairly constant across pharmacotherapy levels. Our study population was comprised of a slightly higher proportion of females than males (53.1 vs. 46.9%) and a relatively low proportion of indigenous patients (0.6%).

**Table 2:** Characteristics of older patients with CRD in WA 1992-2006, overall and by pharmacotherapy level

Patient Characteristic	All patients	SABs	Low dose ICS	Medium dose ICS ± LABs	High dose ICS ± LABs ± OS
Number of patients	108,312	29,700	9,378	37,085	32,149
Average years of follow-up	6.0	4.6	6.3	6.1	7.1
Person-years of follow-up	652,273.0	136,498.5	59,041.5	227,039.4	229,693.6
Number of deaths at the end of follow-up	9,943	2,494	823	2,432	4,194
Mortality rate (per 100,000 person-years)	1,524.4	1,827.1	1,393.9	1,071.2	1,826.3
Mean age at start of follow-up	72.7 ± 7.0	74.7 ± 7.6	72.7 ± 6.8	71.4 ± 6.4	72.3 ± 6.7

SABs = Short Acting Bronchodilators

ICS = Inhaled Corticosteroids

LABs = Long Acting Bronchodilators

OS = Oral Steriod

### All-cause Mortality

**Figure 1:**
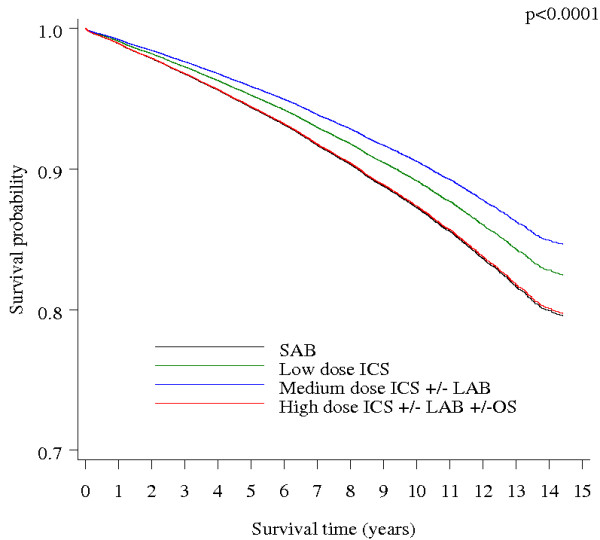
**Adjusted survival curves for all-cause mortality in patients with CRD by pharmacotherapy level**. Curves are adjusted for gender, age start of follow-up, area-based socio-economic status, residential remoteness, the Charlson index of co-morbidity, and indigenous status. SABs = Short Acting Bronchodilators, ICS = Inhaled Corticosteroids, LABs = Long Acting Bronchodilators, OS = Oral Steriods.

Figure 1 shows adjusted survival curves for all-cause mortality in patients with CRD by pharmacotherapy level. Patients in the lowest and highest pharmacotherapy level groups exhibited relatively poor survival throughout the follow-up period, where 79.5% and 79.7%, respectively, were still alive at the end of follow-up. However, patients in the low to medium pharmacotherapy level groups had better survival with a slightly more gradual decline than the patients in the other two groups (82.5% and 84.6% still alive at the end of follow-up, respectively). These differences in survival between the four pharmacotherapy levels were statistically significant (overall p < 0.0001). Adjusted hazard ratios for the effect of low to high pharmacotherapy level on all-cause death compared with the lowest level (short acting bronchodilators (SABs)) were 0.82 (95% CI 0.76-0.90) for low dose ICS; 0.72 (95% CI 0.68-0.76) for medium dose ICS ± long acting bronchodilators (LABs); and 0.94 (95% CI 0.89-0.99) for high dose ICS ± LABs ± oral steroids (OS).

We assessed the likelihood of death due to any cause in relation to patient characteristics, overall and by pharmacotherapy level (Table 3). Males were at increased risk of all-cause death compared with females (HR 1.72, 95% CI 1.65-1.80). The estimates were modified by pharmacotherapy level, being highest in the highest pharmacotherapy level group (HR 1.97, 95% CI 1.84-2.10). The P-value for interaction between pharmacotherapy level and gender for the effect on all-cause death was significant (0.0003). Indigenous patients were more likely to die than non-indigenous patients overall (HR 1.28, 95% CI 0.94-1.73) and in the lowest, medium and high pharmacotherapy level groups, but none of the hazard ratios was significant. Patients living in the most disadvantaged areas were at increased likelihood of death compared with patients in the least disadvantaged areas (HR 1.16, 95% CI 1.08 - 1.24). This association appeared to be higher in the medium pharmacotherapy level group (HR 1.25, 95% CI 1.09 -1.42), but the test for interaction was not significant (P = 0.1161). Residential remoteness did not appear to affect the risk of death overall and by pharmacotherapy level, with all hazard ratios being mostly weak and consistent with chance variation.

**Table 3:** Association between characteristics of CRD patients and risk of death, overall and by pharmacotherapy level

Patient Characteristic	All patients	SABs	Low dose ICS	Medium dose ICS ± LABs	High dose ICS ± LABs ± OS	P-value for interaction
	HR (95% CI)	HR (95% CI)	HR (95% CI)	HR (95% CI)	HR (95% CI)	
Gender*						
Female	1.00	1.00	1.00	1.00	1.00	
Male	1.72 (1.65-1.80)	1.53 (1.40-1.67)	1.56 (1.35-1.82)	1.64 (1.50-1.79)	1.97 (1.84-2.10)	0.0003
Race*						
Non-Indigenous	1.00	1.00	1.00	1.00	1.00	
Indigenous	1.28 (0.94-1.73)	1.38 (0.79-2.42)	1.06 (0.25-4.58)	1.36 (0.70-2.65)	1.18 (0.74-1.87)	0.8841
Socioeconomic status quintile*						
Least disadvantaged	1.00	1.00	1.00	1.00	1.00	
2^nd ^least disadvantaged	1.03 (0.96-1.10)	1.03 (0.90-1.17)	1.03 (0.81-1.31)	0.99 (0.87-1.14)	1.04 (0.93-1.15)	0.8144
Medium disadvantaged	1.05 (0.98-1.12)	0.99 (0.86-1.13)	1.03 (0.82-1.31)	1.05 (0.91-1.20)	1.07 (0.96-1.19)	0.5340
2^nd ^most disadvantaged	1.06 (0.99-1.14)	0.97 (0.85-1.12)	1.20 (0.96-1.51)	1.06 (0.93-1.22)	1.07 (0.96-1.19)	0.7491
Most disadvantaged	1.16 (1.08-1.24)	1.00 (0.87-1.15)	1.14 (0.91-1.44)	1.25 (1.09-1.42)	1.19 (1.07-1.31)	0.1161
Residential remoteness*						
Major Cities	1.00	1.00	1.00	1.00	1.00	
Inner Regional	0.95 (0.89-1.01)	1.00 (0.88-1.15)	1.04 (0.83-1.32)	0.99 (0.87-1.13)	0.87 (0.79-0.97)	0.2424
Outer Regional	1.00 (0.92-1.07)	1.06 (0.91-1.23)	1.00 (0.76-1.32)	0.97 (0.84-1.13)	0.97 (0.87-1.09)	0.5563
Remote	0.83 (0.70-0.99)	0.88 (0.61-1.26)	0.58 (0.24-1.41)	1.09 (0.81-1.45)	0.70 (0.54-0.90)	0.4457
Very Remote	0.87 (0.58-1.30)	0.49 (0.18-1.36)	0.62 (0.08-4.79)	1.13 (0.56-2.28)	1.01 (0.56-1.84)	0.1967

* Adjusted for Charlson index of co-morbidity and all other variables in the table using Cox multivariate regression.

HR = hazard ratio; 95% CI = 95% confidence interval; SABs = Short Acting Bronchodilators; ICS = Inhaled Corticosteroids; LABs = Long Acting Bronchodilators; OS = Oral Steriod

## Discussion

We investigated the effect of patient characteristics on mortality in patients aged ≥ 65 years with CRD in WA, overall and by pharmacotherapy level. Our main findings included a relatively unfavourable survival of patients using only SABS (i.e. in the lowest pharmacotherapy level group), who experienced a slightly worse survival than the highest pharmacotherapy level group. We also found an increased likelihood of all-cause mortality in males particularly if they were in the highest pharmacotherapy level group.

A strength of this study was its whole-population design. We used data that were routinely collected by the WA and Commonwealth governments to ascertain patients with CRD. The accuracy of the WA administrative data and the record links produced by the WADLS has been found to be exceptionally high [20]. However, as the data were collected for administrative purposes rather than research purposes, some consideration is warranted of the inherent limitations in patient ascertainment. First, our CRD study population included everyone at least 65 years old who had ever had a CRD medication dispensed during a 15 year period and thus the majority of our patient sample was selected on the basis of having had a CRD medication dispensed. This case ascertainment strategy appeared to overestimate the cases of CRD. However, based on data analysed by our group from 12 general practices in WA that included diagnostic information, out of 23,850 asthma/COPD prescriptions prescribed by general practitioners, 92% had asthma and/or COPD as reason for visit or prescription (unpublished data) This result indicates a strong correlation between CRD prescribing practice in WA and CRD diagnosis. Also, out of the 72% of patients selected in our study based on CRD medication use only, 30% were selected based on one single prescription. The majority of these 30%, or 56%, were within the SABs only pharmacotherapy level, which by definition includes patients more likely to require only occational treatment with medications. Thus the majority of our study population had established patterns of medication use indicative of a CRD problem. Consequently, by using this selection strategy we can be reasonably confident that with considerable specificity we have included all CRD cases in the older WA population who have ever seen a doctor for breathing problems. Furthermore, when we restricted our analyses to the patients who were selected based on medication use only and to patients selected based on records from all datasets we found the results led to essentially the same conclusions. It is therefore unlikely that the ascertainment strategy had any meaningful impact on our results.

A second consideration relates to the fact that the PBS data consisted only of records of dispensed drugs that were subsidised by the scheme. The vast majority of Australians over 65 years of age are eligible to receive a government concession card, entitling then to receive large PBS and MBS subsidies on medicines and other services. The coverage by these cards in 2004-5 was 90% at ages 65-75 and 95% at ages 75 + years [25]. We expect that the coverage of older people with CRD has been even higher than this. We thus believe it is safe to assume that a very high proportion of CRD patients in this study were concessional beneficiaries. Hence, this would likely have had no more than limited impact on our study since all inhaled corticosteroids and long-acting beta agonist medications were subsidised under the PBS, and inhaled short-acting beta agonist medications and oral corticosteroids were consistently subsidised for concessional beneficiaries [26]. Another issue concerning the use of PBS records in this study is that it is possible to buy inhaled SABs 'over the counter' from pharmacies in WA, which may make the findings of worse survival in the lowest pharmacotherapy level to appear to be due to less access to care. However, the vast majority of patients in the study would have been concessional beneficiaries. Thus seeing a doctor was free from out-of-pocket expense and there were strong financial incentives for the patients to buy SABs using a doctor's prescription instead of over the counter. In addition, the findings were adjusted for socio-demographic and locational disadvantage. We thus believe that access to care is an unlikely explanation for our findings.

The third possible limitation in this study concerns the inclusion of COPD patients with a mild disease in the highest pharmacotherapy level group. Exacerbations from COPD can occur at any severity and COPD patients may therefore be prescribed oral corticosteroids sporadically at any level of severity. We believe this would not have caused a problem in our study since we included in the highest pharmacotherapy level group those patients who were taking oral corticosteroids at a regular basis, using the criterion: > 1 prescription *per year *of oral steroids. Furthermore, despite exclusive oral steroid users being 51% of the patients in the highest pharmacotherapy level group; after excluding those users from this group, the results from our study remained unaltered.

The final limitation relates to the fact that we were unable to obtain data on smoking exposure in the patients. Smoking is a confounder in this study because it affects mortality in asthma/COPD as well being associated with the patient characteristics. The State and Commonwealth do not collect reliable data on smoking exposure as part of their routine administrative data sets. However, all our results were adjusted for factors such as socio-economic status, residential remoteness, gender, indigenous status, and co-morbidity, which most likely removed the majority of the confounding due to smoking.

We examined the survival outcomes of patients in our study according their level of pharmacotherapy and found that those using only SABs experienced the worst survival. This relatively unfavourable survival of patients not using ICS might reflect sub-optimal treatment in at least a sub-group of patients in this category [4,27-32]. That is, some patients in this group could benefit from an escalation of their therapeutic regime including the addition of ICS. This main finding of this manuscript is particularly interesting given the fact that large randomly controlled trials have had difficulty in establishing a beneficial effect of ICS on survival in COPD patients [19].

We observed that males were almost twice as likely to die from any cause than females. These findings accord with previous studies of older asthma patients [11,12,16] as well as reports on the general population [10]. Further, our results indicate that the poorer survival of males was more marked in the highest pharmacotherapy level group. This may have reflected the tendency of men to seek medical care at a later stage in the disease process than women [10,33]. In fact, despite that women with asthma or COPD account for the majority of emergency department (ED) visits and hospitalisations for asthma [34-39] as well as a large proportion of hospitalisations for COPD [40], women who present to the ED have been found to receive more outpatient care and to have better pulmonary function than men [34]. Hospitalised women with asthma have also been reported to experience less severe asthma than men [35], evidenced by the higher incidence of ED intubation of male respiratory patients [41].

We detected a relatively low proportion of indigenous patients in our study (0.6%), which was only slightly lower than the proportion of indigenous individuals among Western Australians aged 65+ years (0.8%) [42]. Indigenous Australians experience significantly decreased life-expectancy and far higher mortality than other Australians [10]. This was evident in our study of older patients with CRD, although the hazard ratio was not statistically significant, which may be due to small numbers. However, our results most likely reflect the persisting gap in access to adequate primary health care that indigenous people are faced with in Australia [43]. For reasons such as cost, distance and lack of transport, indigenous people do not always seek medical assistance when needed [43]. This can mean that any existing disease is more likely to become severe.

## Conclusions

We studied the effect of patient characteristics on all-cause mortality in older patients with CRD, overall and by pharmacotherapy level. We found that patients using only SABs experienced the worst survival. These results indicate that some patients with CRD not currently using ICS could possibly benefit from enhancements to their therapeutic regime, but further research is warranted for corroboration. We also found that increased likelihood of all-cause mortality in males was more pronounced if they were in the highest pharmacotherapy level group. Collectively, these findings illustrate the importance of more actively focusing disease management on mild-to-moderate CRD. This has been emphasized by Cranston et al., who suggest that a more fruitful primary care approach is needed for managing mild-to-moderate CRD in Australia since most guidelines are based on moderate-to-severe disease [44]. Our findings are thus important for primary health care policy because they highlight a need to more actively target services at patients with mild-to-moderate CRD so as to prevent disease progression and subsequent mortality.

## Competing interests

The authors declare that they have no competing interests.

## Authors' contributions

KE analysed and interpreted the data and wrote the article. JDE and CDJH designed the study. DBP, FMS, RR, JDE and CDJH contributed to the interpretation of data and revised the manuscript critically. All authors gave final approval for the manuscript to be published.

## Pre-publication history

The pre-publication history for this paper can be accessed here:

http://www.biomedcentral.com/1471-2458/10/385/prepub
